# Histopathological features for coexistent invasive cancer in large colorectal adenomatous polyps

**DOI:** 10.1093/bjsopen/zraa053

**Published:** 2021-06-16

**Authors:** A Emmanuel, A Haji, S Gulati, J Moorhead, S Papagrigoriadis, B Hayee, S Diaz-Cano

**Affiliations:** 1 Department of Colorectal Surgery and King’s Institute of Therapeutic Endoscopy, King’s College Hospital NHS Foundation Trust, London, UK; 2 King’s Institute of Therapeutic Endoscopy, King’s College Hospital NHS Foundation Trust, London, UK; 3 Department of Histopathology, King’s College Hospital NHS Foundation Trust, London, UK

## Abstract

**Background:**

Histopathological features associated with coexistent invasive adenocarcinoma in large colorectal adenomas have not been described. This study aimed to determine the association of histopathological features in areas of low-grade dysplasia with coexistent invasive adenocarcinoma.

**Methods:**

High-grade lesions (containing high-grade dysplasia or adenocarcinoma) from a cohort of large (at least 20 mm) colorectal adenomas removed by endoscopic resection were subjected to detailed histopathological analysis. The histopathological features in low-grade areas with coexistent adenocarcinoma were reviewed and their diagnostic performance was evaluated.

**Results:**

Seventy-four high-grade lesions from 401 endoscopic resections of large adenomas were included. In the low-grade dysplastic areas, a coexistent invasive adenocarcinoma was associated significantly with a cribriform or trabecular growth pattern (*P* < 0.001), high nuclear grade (*P* < 0.001), multifocal intraluminal necrosis *(P < *0.001), atypical mitotic figures (*P* = 0.006), infiltrative lesion edges (*P* < 0.001), a broad fibrous band (*P* = 0.001), ulceration (*P* < 0.001), expansile nodules *(P < *0.001) and an extensive tumour-infiltrating lymphocyte pattern (*P* = 0.04). Lesions with coexistent invasive adenocarcinoma harboured at least one of these features. The area under the receiver operating characteristic curve (AUROC) for coexistent invasive adenocarcinoma, using frequencies of adverse histopathological factors in low-grade areas, was 0.92. The presence of two or more of these adverse histopathological features in low-grade areas had a sensitivity of 86 per cent and a specificity of 84 per cent for coexistent invasive adenocarcinoma.

**Conclusion:**

Several histopathological features in low-grade dysplastic areas of adenomas could be predictive of coexistent adenocarcinoma.

## Teaser

Histopathological features associated with coexistent invasive adenocarcinoma in large colorectal adenomas have not been described. This study found several histopathological features identified in low grade areas of large adenomas were predictive of the presence of coexistent invasive cancer elsewhere in the lesion. An increasing number of these features is a strong predictor of coexistent invasive adenocarcinoma with an AUROC of 0.92 and ≥2 of these adverse histopathological features in low-grade areas had a sensitivity of 86% and specificity of 84% for coexistent invasive adenocarcinoma.

## Introduction

Advances in endoscopic diagnosis and resection techniques, together with an agreement on standardized classifications of colorectal tumour morphology, have led to the widespread adoption of organ-conserving endoscopic treatment of superficial colorectal neoplastic tumours in many expert centres^1^^,^^2^. However, the risk of incorrectly treating tumours harbouring invasive carcinoma remains a significant concern of even expert interventional endoscopists.

Reliable and reproducible endoscopic or histopathological features predicting the risk of an adenomatous polyp containing invasive cancer are lacking^3^. Although the varying risk of covert invasive cancer in any gross morphological subtypes of large colorectal superficial neoplastic tumours is now recognized, many, if not most, invasive cancers are unexpected occurrences in large adenomas^4–6^. As a result of this uncertainty, biopsy sampling is common practice in many institutions, despite being of limited use in predicting invasive cancer and largely discouraged in guidelines^7^^,^^8^. However, on histopathological assessment, large colorectal superficial neoplastic lesions typically comprise areas of variable architecture and cytology, and areas of invasion are small relative to the size of the lesion. Detailed reports on the histopathological features of large colorectal adenomas subjected to endoscopic resection are lacking, and there are no reports on features of the low-grade areas of adenomatous polyps associated with coexistent invasive cancer in the lesion. Knowledge of such features could help with interpretation of biopsy samples, help decision-making where there is diagnostic doubt because of piecemeal resection or incomplete sample retrieval, or inform decisions regarding surveillance intervals.

This study aimed to determine the association of histopathological features in areas of low-grade dysplasia with coexistent invasive adenocarcinoma in large (at least 20 mm) high-grade adenomatous polyps removed by endoscopic resection, and assess the diagnostic value of these associations.

## Methods

Data from a prospectively maintained database of endoscopic resections of large (20 mm or above) colorectal superficial neoplastic lesions performed between January 2011 and September 2016 were analysed. Approval for this study was granted by the Health Research Authority, UK.

### Setting and procedures

King’s College Hospital NHS Foundation Trust in London is a regional tertiary referral centre for advanced endoscopic resection of large colorectal superficial neoplastic lesions and early cancer. Patients were referred when the endoscopist performing the index procedure felt the lesion was beyond the capabilities of the referring institution because of considerable size, difficult location, challenging morphology or previous failed attempts at resection, or early cancer was suspected for consideration of local excision.

The approach to assessment and resection has been described previously^9^^,^^10^. All lesions were assessed before resection with chromoendoscopy using indigo carmine and magnification colonoscopy (colonoscopes: CF-H260AZL (Olympus, Tokyo, Japan) and EC-600Z (Fujifilm, Düsseldorf, Germany)) and classified according to Kudo pit pattern and vascular pattern (Showa classification)^11^^,^^12^. Selected lesions were also evaluated with variable high-frequency mini-probe ultrasound (Fujifilm) to test for submucosal invasion. The techniques used for resection included endoscopic submucosal dissection (ESD), hybrid ESD, and endoscopic mucosal resection (EMR) or piecemeal EMR (pEMR). Patients underwent surveillance endoscopy at 3–6 and 12 months, and, if no recurrence was detected, colonoscopy at 36 months.

The histopathological features of resection specimens were reported by specialist gastrointestinal pathologists, and cases were discussed in a multidisciplinary colorectal tumour meeting to confirm management.

### Case selection

Lesions were included when a finding of high-grade dysplasia or adenocarcinoma was recorded by the reporting pathologist. Lesions were excluded when only low-grade dysplasia was recorded or no areas of high-grade dysplasia or adenocarcinoma were found on detailed specimen review.

### Histopathological analysis

All detailed histopathological evaluations for this study were performed by a single expert gastrointestinal histopathologist. Haematoxylin and eosin-stained slides from 4-µm sections of formalin-fixed paraffin-embedded tumour tissue blocks of the entire lesion were examined with light microscopy.

Detailed histopathological evaluation included an assessment of the architectural and cytological features in the majority of low-grade dysplastic areas of the lesion and evaluation of the high-grade regions. Evaluation of the low-grade dysplastic regions comprised assessment of the predominant and any secondary growth patterns (tubular/villous or cribriform), quantification of the proportion of tubular and villous architecture, predominant nuclear grade (high or low grade), presence of atypical mitotic figures, glandular intraluminal necrosis, ulceration/mucosal break, growth pattern at the lesion edge (pushing or infiltrative), presence of a broad fibrous band, and the pattern of tumour-infiltrating lymphocytes (interstitial, interface, or both).

High-grade areas included high-grade dysplasia, intramucosal adenocarcinoma or invasive adenocarcinoma. Intramucosal adenocarcinoma was differentiated from high-grade dysplasia based on lamina propria invasion, cribriform growth pattern or solid areas, intraluminal necrosis and high-grade nuclear atypia with loss of polarity. Although many Western pathologists do not recommend use of the term intramucosal cancer, as it can be differentiated morphologically from high-grade dysplasia and shares many of these features in common with invasive cancer, it was included for the purposes of this study. Invasive adenocarcinoma was defined as invasion through the muscularis mucosa. Detailed histopathological evaluation of high-grade areas included the size of the dominant area measured in millimetres, multifocality, nuclear grade, atypical mitoses, and expansile nodules.

It is important to note that, in keeping with accepted histopathology reporting guidelines, the diagnosis of high-grade dysplasia or intramucosal cancer requires characteristic cytological features on a background of clear architectural abnormalities involving more than two glands. Furthermore, for a diagnosis of high-grade dysplasia, the presence of a combination of these features is required. However, it is well recognized that isolated cytological or architectural features, or features involving fewer than two glands, not amounting to a diagnosis of high-grade dysplasia, may occur in low-grade dysplastic areas, and it was according to these criteria that such features were identified in low-grade dysplastic areas (but not fulfilling criteria for high-grade dysplasia/intramucosal cancer) in this study^13^^,^^14^.

### Statistical analysis

Results were reported using mean(s.d.) values for continuous variables and frequencies for categorical variables. Associations between histopathological features in low-grade areas with coexistent adenocarcinoma were evaluated using the χ^2^ test. Area under the receiver operating characteristic (ROC) curve (AUROC), sensitivity and specificity were used to test the diagnostic performance of the number of any significantly associated histopathological features with the presence of invasive adenocarcinoma. To ascertain the importance of the association of individual morphological features with invasive cancer, and to gain an understanding of the cumulative changes associated with malignant change, the perspective of a null hypothesis to promote specificity (absence of the test variable in case of no malignancy) was used to calculate the probability of the presence of each variable in the absence of coexistent invasive cancer. Statistical analyses were performed using Epi Info version 7 (Centers for Disease Control, Atlanta, GA, USA) and SPSS^®^ version 26 (IBM, Armonk, New York, USA).

## Results

Over the study period, endoscopic resection was performed for 401 large (at least 20 mm) colorectal superficial neoplastic lesions (*Table 1*) with a mean(s.d.) size of 55.6(30.3) mm (median 50, range 20–160 mm) using endoscopic mucosal resection or pEMR (*n* = 319), or ESD or hybrid ESD (*n* = 82). The patients were predominantly men (223, 55.6 per cent), and the mean age was 72 years. The vast majority of lesions were subtypes of laterally spreading tumour.

**Table 1:** Characteristics of patient and lesions

	No. of patients* (*n*=401)
**Age (years)†**	72(11.3) (33–94)
**Sex ratio (M : F)**	223 : 178
**Size of lesion (mm)†**	55.6(30.3) (20–160)
**Location**	
Right colon	140 (33.9)
Left colon	105 (26.2)
Rectum	156 (38.9)
**Morphology**	
Is/Isp	75 (18.7)
LST, granular homogeneous	205 (51.1)
LST, granular mixed-nodular	107(26.7)
LST, non-granular	14 (3.5)
**Kudo pit pattern‡**	
I–II	7 (1.7)
III–IV	374 (93.3)
Vi or Vn	20 (5.0)
**Vascular pattern‡**	
Faint/normal	8 (2.0)
Network/dense	386 (96.0)
Irregular/sparse	8 (2.0)
**Resection technique**	
EMR/pEMR	319 (79.6)
ESD/hybrid ESD	82 (20.4)
Histology	
Non-neoplastic	7 (1,7)
Sessile serrated lesion/adenoma	19 (4.7)
Adenoma	341 (85.0)
Adenocarcinoma	25 (6.2)
Missing	9 (2.2)
**Tissue sampling/biopsy before referral**	332 (82.8)

*With percentages in parentheses unless indicated otherwise; †values are mean(s.d.) (range). ‡Most dysplastic area recorded. Is/Isp, sessile/sub-pedunculated; LST, laterally spreading tumour; EMR, endoscopic mucosal resection; pEMR, piecemeal EMR; ESD, endoscopic submucosal dissection.

Invasive adenocarcinoma was reported in 6.2 per cent, and 82.8 per cent had been subjected to tissue sampling/biopsy before referral.

### High-grade lesions

Of the 401 large colorectal lesions treated by endoscopic resection, 90 were reported to harbour either invasive adenocarcinoma or high-grade dysplasia and were selected for detailed histopathological assessment. Records could not be obtained for four cases, haematoxylin and eosin-stained tissue sections were missing or incomplete for eight lesions, and four were deemed to be low-grade lesions after detailed evaluation and were excluded. Detailed histopathological evaluation of the remaining 74 lesions is thus reported.

Coexistent invasive adenocarcinoma was encountered in 29 lesions (39 per cent) and coexistent intramucosal adenocarcinoma in 10 other lesions (14 per cent). The remainder harboured high-grade dysplasia.

**Fig. 1:**
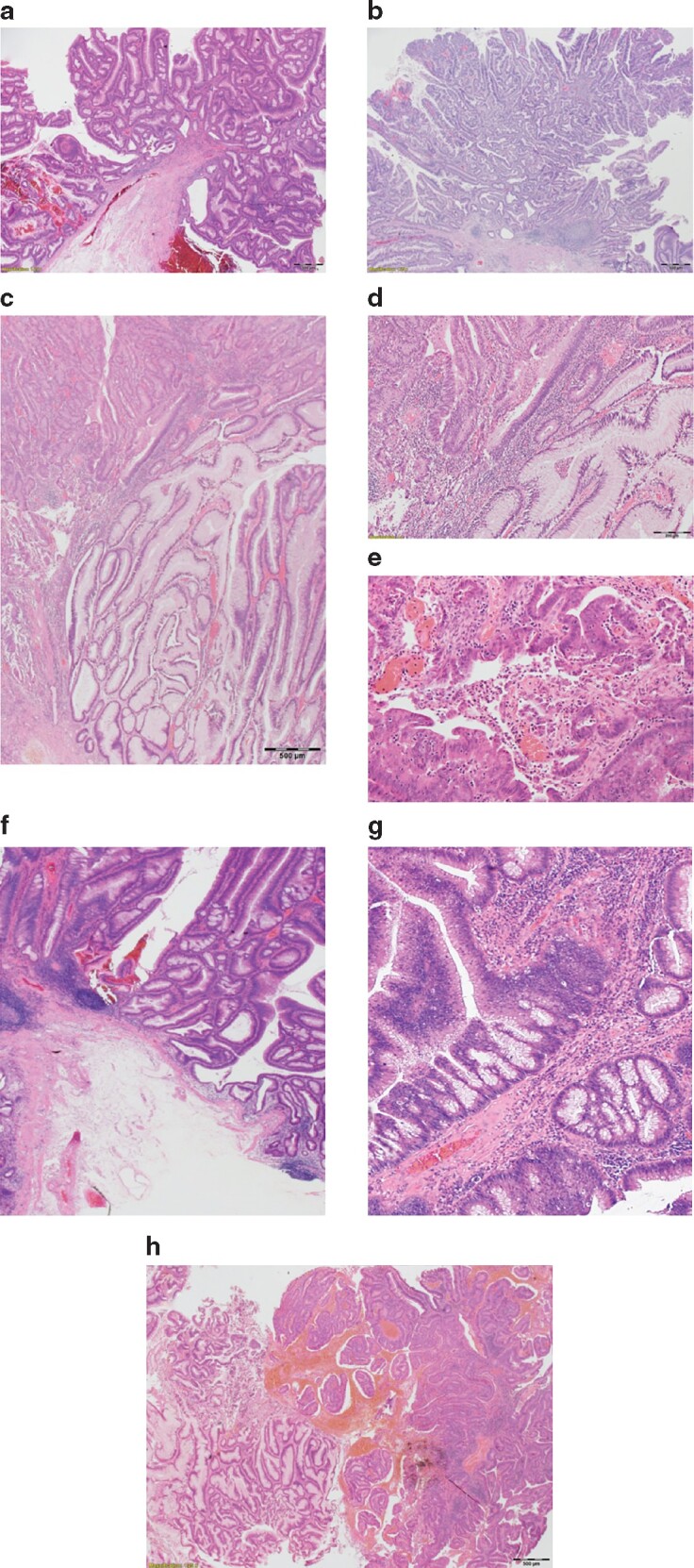
Examples of adverse histopathological factors identified in low-grade dysplastic areas of large adenomas containing high-grade dysplasia or invasive cancer **a,b** Lesion edges in low-grade dysplasia. Adenomatous polyps can reveal **a** pushing or b infiltrative edges in the low-grade dysplastic areas (haematoxylin and eosin stain, magnification 13×). The lesion edges appear smooth and well defined in the pushing edges, and isolated glands are identified at the front in infiltrative lesions. **c–e** Lymphocytic infiltrate with an interstitial pattern. The inflammatory cells frequently infiltrate irregularly between the glands, partially obscuring the glandular outline of lesions with infiltrative inflammation pattern (**c** haematoxylin and eosin stain, magnification 13×; **d** haematoxylin and eosin stain, magnification 40×). In this pattern, the lymphocyte exocytosis (intraepithelial migration of mature lymphocytes) is a common finding (**e** haematoxylin and eosin stain, magnification 100×). **f–g** Lymphocytic infiltrate with an interface pattern. The inflammatory infiltrate concentrates at the basal interface between the lesion and the surrounding normal tissue of lesions with interface inflammation pattern (haematoxylin and eosin stain, magnification 13× (**f)** and 40× (**g)**). **h** Variable growth pattern in areas of low-grade dysplasia. The most common growth pattern in low-grade dysplastic areas is tubular and papillary (left side of image), followed by the trabecular pattern in heavily inflamed areas (right side of image) (haematoxylin and eosin stain, magnification 13×).

*Table 2* shows an exploratory univariable analysis of histopathological factors in the low-grade dysplastic areas of lesions associated with either coexistent invasive adenocarcinoma or intramucosal adenocarcinoma. *Fig. 1* gives examples of selected adverse histopathological features identified in low-grade dysplastic areas.

**Table 2:** Exploratory univariable analysis of histopathological morphology and cytology factors in low-grade dysplastic areas potentially associated with coexistent invasive adenocarcinoma or intramucosal adenocarcinoma in high-grade lesions after detailed histopathological assessment

	Coexistent invasive adenocarcinoma (*n*=29)	*P*	Coexistent intramucosal adenocarcinoma (*n*=10)	*P*
**Cribriform/trabecular growth pattern**				
Present	15	<0.001	3	0.153
Absent	14		7	
**Villous component (%)**				
≥30	4	0.507	1	0.889
<30	25		9	
**Nuclear grade**				
High	14	<0.001	2	0.055
Low	15		8	
**High-grade dysplasia**				
Multifocal	16	0.825	9	0.019
Focal	13		1	
**Intraluminal necrosis**				
Multifocal	25	<0.001	6	<0.001
Focal or none	4		4	
**Atypical mitotic figures**				
Present	8	0.006	0	0.589
Absent	21		10	
**Lesion edges**				
Infiltrative	12	<0.001	0	1.000
Pushing	17		10	
**Broad fibrous band present**				
Present	6	0.001	0	1.000
Absent	23		10	
**Ulceration**				
Present	13	<0.001	2	0.055
Absent	16		8	
**Expansile nodule**				
Present	15	<0.001	1	0.632
Absent	14		9	
**Tumour-infiltrating lymphocyte pattern**				
Interstitial	16	0.040	4	0.491
Interface only	13		6	

Coexistent invasive adenocarcinoma was associated significantly with the presence, in low-grade dysplastic areas, of any cribriform or trabecular growth pattern (*P* < 0.001), high nuclear grade (*P* < 0.001), multifocal intraluminal necrosis (*P* < 0.001), atypical mitotic figures (*P* = 0.006), infiltrative lesion edges (*P* < 0.001), a broad fibrous band *(P = *0.001), ulceration (*P* < 0.001), expansile nodules *(P < *0.001) and a more extensive tumour-infiltrating lymphocyte pattern (*P* = 0.040). Multifocal high-grade dysplasia and a more extensive villous component were not associated with coexistent invasive adenocarcinoma. Of the 29 lesions with coexistent invasive cancer, 7 had no areas of high-grade dysplasia and 13 had only focal high-grade dysplasia. In contrast, coexistent intramucosal adenocarcinoma was significantly associated only with multifocal high-grade dysplasia (*P* = 0.019) and multifocal intraluminal necrosis (*P* < 0.001).

The histopathological factors in low-grade dysplastic areas associated with invasive adenocarcinoma frequently occurred together, such that all lesions with coexistent invasive adenocarcinoma harboured at least one of these morphological or cytological features and only 4 of 29 had only one feature. Coexistent adenocarcinoma was increasingly more likely with the presence of multiple features (*Table 3*). The AUROC for coexistent invasive adenocarcinoma, using varying frequencies of adverse histopathological factors in low-grade dysplastic areas, was 0.92 (95 per cent c.i. 0.86 to 0.98). The presence of two or more of these adverse histopathological features in low-grade dysplastic areas had a sensitivity of 86 (95 per cent c.i. 69 to 95) per cent and a specificity of 84 (71 to 92) per cent for the diagnosis of coexistent invasive adenocarcinoma.

**Table 3:** Cumulative frequency of occurrence of adverse histopathological factors in low-grade dysplastic areas in relation to presence of coexistent adenocarcinoma

	No. of adverse histopathological factors in low-grade dysplastic area									
	0	1	2	3	4	5	6	7	8	9
**Coexistent invasive adenocarcinoma**										
Present	0	4	4	4	7	1	2	2	4	1
Absent	19	19	3	3	1	0	0	0	0	0
**True positive rate**	1.000	1.000	0.862	0.724	0.586	0.345	0.310	0.241	0.172	0.035
**False positive rate**	1.000	0.578	0.156	0.089	0.022	0.000	0.000	0.000	0.000	0.000

From a diagnostic perspective, the predictive value of a coexistent invasive adenocarcinoma with two variables ranged from 78 per cent (using broad fibrous bands and lesion edges or atypical mitoses) to 91 per cent (using multifocal high-grade dysplasia and tumour-infiltrating lymphocyte pattern). The predictive value increased when more variables were added. For three variables, it ranged from 90 per cent (using broad fibrous bands, lesion edges, and atypical mitoses) to 96 per cent (adding the cribriform pattern to the best two used above). When four or more variables were used, the predictive value plateaued to 96–98 per cent or better.

**Fig. 2:**
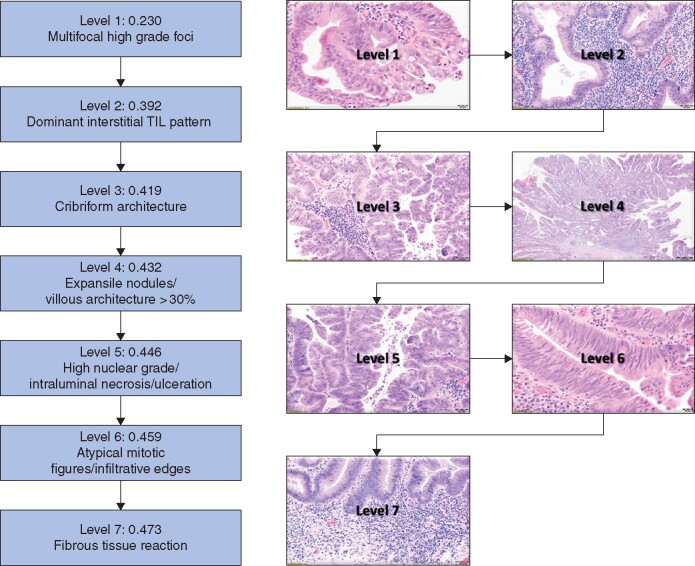
Morphological variables predictive of malignancy in adenomatous polyps Histological findings able to predict malignancy can be stratified in several levels according to their statistical weight. This systematic evaluation allows a practical approach to ascertain the cumulative changes associated with malignant transformation. TIL, tumour-infiltrating lymphocyte.

After applying a null hypothesis that promoted specificity (absence of the test variable in cases with no invasive cancer), the histological variables revealed different statistical weight and were categorized in seven levels for predicting coexistent invasive cancer (*Fig. 2*). This approach allows a sequential and systematic application of the results, and confirmed the requirement of accumulating coexistent histological variables for a reliable diagnosis.

## Discussion

Several architectural and cytological features present in the low-grade dysplastic areas of adenomatous polyps are strongly associated with coexistent invasive adenocarcinoma elsewhere in the lesion. It is, therefore, possible to predict invasive malignancy by detailed histopathological evaluation of large (20 mm or more) colorectal adenomatous polyps removed by endoscopic resection. These features are absent from the low-grade dysplastic areas of lesions without invasive adenocarcinoma, even those harbouring areas consistent with intramucosal adenocarcinoma. They occur in combination where there is coexistent invasive adenocarcinoma.

This study has provided insight into the increasing probability of coexistent invasive cancer with accumulating adverse features in the low-grade dysplastic areas, and suggests the potential nature of progression of lesions toward high-grade or invasive tumours.

There are significant potential applications of these findings to contemporary clinical practice. Considerable recent progress has been made recently towards standardizing classifications of morphology and surface characteristics of superficial colorectal tumours^2^^,^^11^^,^^12^. It has been well established, initially from large Japanese series and subsequently confirmed in Western series, that the risk of so-called ‘covert’ invasive cancer in large adenomas varies considerably according to these standardized lesion morphologies^4^^,^^5^. Assessment of surface characteristics such as Kudo pit pattern and vascular patterns, primarily using magnification endoscopy, are also useful in predicting the risk of coexistent invasive adenocarcinoma or high-grade lesions^14^. However, despite the current increasing use of advanced endoscopic resection to treat large colorectal superficial neoplastic lesions in preference to radical surgery, the skills amongst general endoscopists for advanced lesion assessment and characterization of the risk of an adenoma harbouring invasive cancer are still somewhat lacking in Western practice^15^. As a result, routine biopsy sampling and occasionally injudicious attempts at resection are widespread—performed for at least 80 per cent of lesions in the present authors’ experience^16^. Although the decision regarding resection technique for superficial colorectal tumours should be based on advanced endoscopic assessment, the results of the present study could help with decision-making in difficult situations, for example where lesions have undergone piecemeal resection without retrieval of all resected tissue for histopathological analysis, or where injudicious piecemeal resection has resulted in a problematic accurate histopathological assessment of a high-grade lesion.

Some studies have found an association between the presence of high-grade dysplasia or intramucosal cancer and coexistent invasive cancer in colorectal adenomas, and others have suggested that the finding of high-grade dysplasia on biopsy samples is predictive of coexistent invasive adenocarcinoma^17^^,^^18^. However, biopsy involves sampling small amounts of tissue from large lesions, and the chance of obtaining tissue from an area of high-grade dysplasia in such large lesions is low. Targeting an area of high-grade dysplasia seems even more unlikely: despite this being a selected set of high-grade lesions, 7 of 29 lesions with coexistent invasive cancer had no areas of high-grade dysplasia, and 13 per cent of all lesions that did contain high-grade dysplasia had only focal high-grade dysplasia. This explains partly why biopsy sampling showing high-grade dysplasia is unreliable for diagnosing coexistent invasive adenocarcinoma.

The several adverse histopathological factors identified in this study in low-grade dysplastic areas, which account for most of the tissue in large colorectal adenomas, could provide a more accurate prediction of coexistent invasive adenocarcinoma. These features are identifiable using standard histopathological techniques examining haematoxylin and eosin-stained slides, and are within the scope of practice of any specialist gastrointestinal pathologist without the need to resort to specialized staining techniques or other specialist methodologies.

Intramucosal adenocarcinoma, as defined in this study, has almost identical morphological and cytological characteristics to those of invasive adenocarcinoma, aside from invasion through the muscularis mucosa. Similar to coexistent invasive adenocarcinoma, coexistent intramucosal adenocarcinoma was associated significantly with multifocal intraluminal necrosis in the low-grade dysplastic areas and was far more frequently associated with high nuclear grade and ulceration, which both closely approached statistical significance. However, in contrast to invasive adenocarcinoma, it was not associated with any of the other adverse histopathological features in low-grade dysplastic areas, including any cribriform growth pattern and a large tumour-infiltrating lymphocyte pattern.

The study did not include lesions composed entirely of low-grade areas without foci of high-grade dysplasia as these, in many ways, represent a different cohort without diagnostic or assessment challenges. A significant association of several adverse factors with coexistent invasive cancer and an association with an accumulating frequency of these factors in invasive cancer was documented. Importantly, these features were absent in lesions that did not harbour invasive cancer. Therefore, it is unlikely that an extensive examination of entirely low-grade lesions would alter the significant findings of this study.

This research has some limitations. Detailed histopathological evaluation was performed by a single expert gastrointestinal histopathologist. Although some interobserver discrepancies are part of most histopathological assessments^19^, the application of clear and simple definitions of the variables in this study limits this possibility. In addition, assessment by a single expert in the present study resulted in consistency of reporting across all cases. Reproducibility of histopathology findings is improved by reducing the available categories, and the reproducibility of a two-tier grading system is much more consistent than that of a three-tier system^20^^,^^21^. As a result, a two-tier system has been adopted in the dysplasia grading for the bowel cancer screening programme in the UK^13^. Possibly, by adopting a two-tier grading system in the present study reproducibility of the findings by other pathologists will be improved. In general, as the main area of disagreement among pathologists is the distinction between mild and moderate dysplasia, the reproducibility could be achieved nevertheless. The histological variables as applied in this study would not be expected to cause a significant problem for any certified pathologist to apply these findings reliably, and were assessed on standard haematoxylin and eosin-stained sections with no other specialist techniques. Furthermore, the present study design was not intended to test for interobserver agreement; rather, the aim was to investigate the association of histopathological changes that might predict the presence of coexistent invasive adenocarcinoma.

Several histopathological features present in low-grade dysplastic areas of large colorectal adenomatous polyps are predictive of coexistent malignancy. The presence of multifocal high-grade dysplasia, interstitial tumour-infiltrating lymphocytes and a cribriform pattern is useful for that task, requiring at least two of the high-risk histological variables for a reliable prediction of coexistent invasive cancer in this series. The cumulative presence of adverse features in areas of low-grade dysplasia progressively improves the diagnostic value. They occur in several combinations, and an increasing number of these features in low-grade dysplastic areas is a strong predictor of coexistent invasive adenocarcinoma with an AUROC of 0.92. Provided that sufficient material is available for evaluation, these findings will help direct accurate assessment of large biopsy samples or piecemeal resections to decide on future management.

## Disclosure

The authors declare no conflict of interest.
